# Condition Monitoring of Wind Turbine Systems by Explainable Artificial Intelligence Techniques

**DOI:** 10.3390/s23125376

**Published:** 2023-06-06

**Authors:** Davide Astolfi, Fabrizio De Caro, Alfredo Vaccaro

**Affiliations:** 1 Department of Engineering, University of Perugia, Via G. Duranti 93, 06125 Perugia, Italy; davide.astolfi.green@gmail.com; 2 Department of Engineering, University of Sannio, Piazza Roma 21, 82100 Benevento, Italy; fdecaro@unisannio.it

**Keywords:** wind energy, wind turbines, explainable artificial intelligence, data analysis, condition monitoring, SCADA

## Abstract

The performance evaluation of wind turbines operating in real-world environments typically relies on analyzing the power curve, which shows the relationship between wind speed and power output. However, conventional univariate models that consider only wind speed as an input variable often fail to fully explain the observed performance of wind turbines, as power output depends on multiple variables, including working parameters and ambient conditions. To overcome this limitation, the use of multivariate power curves that consider multiple input variables needs to be explored. Therefore, this study advocates for the application of explainable artificial intelligence (XAI) methods in constructing data-driven power curve models that incorporate multiple input variables for condition monitoring purposes. The proposed workflow aims to establish a reproducible method for identifying the most appropriate input variables from a more comprehensive set than is usually considered in the literature. Initially, a sequential feature selection approach is employed to minimize the root-mean-square error between measurements and model estimates. Subsequently, Shapley coefficients are computed for the selected input variables to estimate their contribution towards explaining the average error. Two real-world data sets, representing wind turbines with different technologies, are discussed to illustrate the application of the proposed method. The experimental results of this study validate the effectiveness of the proposed methodology in detecting hidden anomalies. The methodology successfully identifies a new set of highly explanatory variables linked to the mechanical or electrical control of the rotor and blade pitch, which have not been previously explored in the literature. These findings highlight the novel insights provided by the methodology in uncovering crucial variables that significantly contribute to anomaly detection.

## 1. Introduction

The generation of electricity from renewable sources has been rapidly increasing in recent years, with an estimated growth from 10.8% in OECD countries in 2019 to one-third by 2035 [1]. Among renewable energy technologies, wind energy is considered to be a leading contender for the coming decades, owing to its favorable energy density and conversion efficiency. The global installation of wind turbines nearly doubled from 58 GW in 2019 to 111 GW in 2020 [2], and this trend has been further accelerated by recent geopolitical trends.

One of the keystones in enhancing the profitability of wind energy is the containment of operation and maintenance (O&M) costs, which can account for the 30% of the total costs over the lifetime of a wind plant [3], and can dramatically increase for offshore installations. This underscores the increasing attention being given to wind turbine condition monitoring [4], which is a challenging task due to the complex nature of wind turbines and their exposure to nonstationary operation conditions.

Supervisory control and data acquisition (SCADA) systems have traditionally been utilized for the remote control of wind turbines, but their concept has evolved to become a powerful tool for condition monitoring [5,6]. The basic idea is to measure selected channels at an appropriate sampling rate (ranging from one second for wind speed to several seconds for nacelle position) and store them after averaging over a standardized time basis, typically ten minutes. Even with the limited number of measurement channels collected by wind turbines in the 600–850 kW era, the use of SCADA data for condition and performance monitoring has become standard practice, with the power curve analysis even being standardized by the International Electrotechnical Commission (IEC) [7]. The power curve, which relates the input wind speed to the output power, is widely recognized as the most intuitive representation of wind turbine performance [8,9,10]. In this regard, the IEC has provided guidelines for standardizing, renormalizing, and analyzing power curve data. However, modern wind turbines, with rated powers ranging from 4 to 7 MW, are equipped with hundreds of measurement channels that capture various aspects, including environmental conditions, mechanical response (temperatures and vibrations), electrical behavior, and wind turbine control, among others. While this increases the potential of SCADA-based condition monitoring for wind turbine systems, it also presents new challenges.

The analysis of wind turbine power curves, following the guidelines of the IEC, has its limitations. It can tell us when a turbine is underperforming by comparing it to a reference, but it falls short in explaining the underlying causes. Recent breakthroughs in the field have sparked interest in developing multivariate data-driven models for power curves, aiming to overcome this limitation [11,12]. These innovative approaches consider the output power of wind turbines as a function of multiple input variables or covariates to provide a more comprehensive understanding of the factors that influence wind turbine performance. However, as discussed in more detail in Section 1.2, most of the studies that employ multiple input–single output models are black-box models, which can be opaque, especially as the number of input variables increases. Therefore, there are open issues related to explainability in multivariate power curve models, which can be summarized as follows:

- How to rigorously select the covariates to include in the model;
- How to attribute an operational anomaly to specific covariates potentially associated with a certain subsystem.

Therefore, the objective of this study is to address the above two open questions by utilizing explainable artificial intelligence (XAI) techniques in the analysis of two real-world test cases. The advancements in XAI [13,14] offer promising potential for the proper selection and interpretation of input variables, as XAI methods can provide insights into the relative importance of different sensors in determining a certain output, such as wind turbine power. In light of this, recent related work is reviewed in Section 1.1.

### 1.1. Related Work

A multivariate data-driven wind turbine power curve model is a regression with multiple input variables and one output (the power). However, there are several open issues and no consolidated standards regarding the selection of input data and the model structure. As a result, there have been numerous recent studies in the literature addressing these challenges.

In the literature (e.g., [15]), the use of meteorological mast data, such as air density, humidity, turbulence intensity, and wind shear, as covariates in addition to wind speed is a minority approach, as it has been established that operational variables are more appropriate as additional covariates [16]. However, a recent study by [17] utilizes multivariate polynomial regressions to model wind turbine power curves based on wind speed and turbulence measurements obtained from nacelle lidars. Another seminal study in the literature is [18], where only data collected from the SCADA control system are employed, but additional covariates such as ambient temperature and wind direction are considered. A similar approach is pursued in [19], where the power of a wind turbine is modeled as a function of various SCADA-collected environmental variables, including wind speed, temperature, turbulence intensity, and wind direction, by comparing several models, such as the Gaussian mixture copula model and regressive and Bayesian artificial neural networks.

A well-established study on the use of SCADA-collected operation variables for multivariate data-driven power curves is presented in [20], where variables such as wind direction, yaw error, blade pitch, and rotor speed are employed in addition to wind speed. Blade pitch and rotational speed are the most commonly used additional covariates in the literature [12,21], as they are fundamental parameters that influence the power coefficient of a wind turbine, which in turn depends on the tip–speed ratio (equivalent to rotational speed) and blade pitch, as dictated by first principles [22].

Some recent studies have addressed the problem of multivariate modeling of wind turbine power by segmenting the data based on various criteria. For instance, in [23], a piecewise multivariate Gaussian Process (GP) model is proposed, where different models are created for each wind speed bin, incorporating environmental (e.g., wind speed and direction), operational (e.g., rotational speed and blade pitch angle), and thermal variables (e.g., gear bearing temperature, gear oil temperature) as input variables. A similar approach of segmenting the wind turbine power curve and constructing multivariate models for each segment is also explored in [11,24]. Another study by [25] focuses on accounting for the effect of yaw misalignment on wind turbine power, and therefore, the data are segmented per bin of yaw angle, with a yaw-adjusted additive multivariate kernel (AMK) model established for each segment. For a recent review on data-driven wind turbine power curve modeling, refer to [26].

A few recent studies, such as [27,28], have focused on automatic feature selection from a large set of potential covariates. In particular, they unravel the influence of wind turbine models, specifically focusing on the type of control employed, whether it be electrical or hydraulic blade pitch control. Their findings shed light on the nuanced relationship between the choice of features and the turbine model type, further fueling our quest to push the boundaries of knowledge in this field. Building upon this insight, the objective of our study is to further advance the field by formulating XAI methods for feature selection and interpretation of the constructed model and applying them for anomaly detection in wind turbine power curve modeling.

### 1.2. Contributions of the Work

The objective of the present work is to advance the state of the art in multivariate wind turbine power curve modeling and its application. This will be achieved through several perspectives, including:

- Enhancing model accuracy.
- Gaining insight into feature selection and improving explainability.
- Applying the model for condition monitoring purposes.

A comprehensive examination of features is conducted using explanatory methods, including a sequential feature selection (SFS) algorithm and the calculation of Shapley coefficients [29]. The selected regression model is GP, which is widely used in the literature for such applications [12,30]. Notably, the methodology proposed in this work provides a replicable criterion for selecting input variables (SFS) and quantifying the impact of each variable on the average error metrics (Shapley coefficients), which is particularly relevant for a black-box model such as GP.

A distinctive feature of this study is the broader set of considered possible covariates compared to the existing literature, including previously unexplored variables. The variable set includes measurements related to rotor control (rotational speed and hydraulic or electrical blade pitch) as well as the mechanical and thermal behavior of the wind turbine, based on the authors’ expertise. A large amount of real data, with different types of technology (hydraulic vs. electrical blade pitch control), are considered, enriching the experimental result section of this manuscript.

In summary, the anticipated innovative results of this work are as follows:

- The optimal selection of input variables is found to be nontrivial and differs from the typical set employed in the literature, which usually includes only blade pitch and rotational speed.
- Specifically, the role of blade pitch as an input variable should be reconsidered if more explanatory input variables are identified.
- The proposed method is demonstrated to be effective for condition monitoring of wind turbine systems, successfully diagnosing three anomalies. Notably, two of these anomalies are related to blade pitch control (hydraulic and electrical, respectively), and one is associated with transverse tower vibrations, likely also influenced by blade pitch behavior.

The proposed application for condition monitoring is particularly advantageous in order to detect hidden anomalies in the operational behavior of wind turbines. Currently, the state of the art for the use of SCADA-collected data for condition monitoring is the normal behavior modeling of certain features, which are selected as targets based on first principles. For example, the temperature increase at meaningful rotating components of the wind turbine is targeted as a symptom of evolving damage. The approach proposed in the present work does not need to know a priori what the target is to monitor for identifying an anomaly. It starts from a set of representative features that is possibly vast, and highlights differences in the operation behavior. This allows for anomalies to be unveiled that are overlooked in the wind farm operation practice and in the scientific literature, such as those highlighted for the test cases of this work.

The rest of the manuscript is structured as follows: Section 2 illustrates the proposed methodology, Section 3 describes data and the experimental setting, and Section 4 describes the conducted experiments. A discussion of the results is presented in Section 5, while Section 6 draws the main conclusions.

## 2. Proposed Methodology

The present study focuses on the construction of a multivariate power curve model using a combination of Gaussian process regression (GPR) and XAI-based methods. The novelty is that the accuracy of the built model, combined with the employment of the Shapley coefficient, allows for the realization of effective condition monitoring systems.

**Figure 1:**
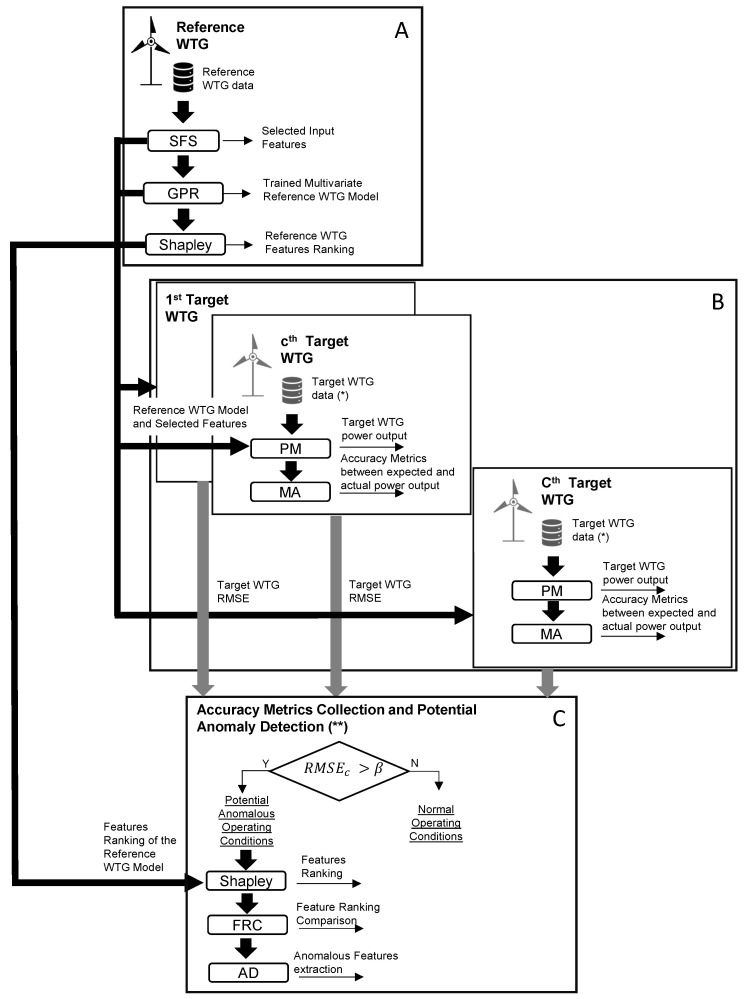
Workflow of the proposed methodology. (*) The selected features are the same extracted from the target WTG. (**) Iteration over each target WTG.

The proposed methodology follows a workflow illustrated in Figure 1 and can be divided into three main blocks, labeled as (A), (B), and (C).

- In block (A), the initial stage involves the collection of data from a reference wind turbine generator (WTG) over a specific time period. To identify the most influential variables from a vast pool of potential inputs, the sequential forward selection (SFS) algorithm is applied. Subsequently, the selected covariates obtained from SFS are utilized to construct a Gaussian process regression (GPR) model. To gain insights into the average error and the contribution of each selected covariate, Shapley coefficients [31,32] are computed.
- The trained model, together with the selected input features and their rankings based on Shapley coefficients, is deployed to each WTG within the wind farm array, denoted as the *c*-th WTG. In block (B), the model trained using the reference WTG is utilized to evaluate the performance of the *c*-th WTG. The input data for the *c*-th WTG are filtered according to the sequential forward selection (SFS) process, ensuring optimal inputs for performance monitoring (PM). By comparing the measured and estimated outputs, accuracy metrics (AM) are computed. Once the operations in block (B) are concluded, an RMSE collection is obtained, consisting of one sample per WTG, providing insights into the overall performance of the wind farm.
- In block (C), leveraging the information gathered from the RMSE distribution, outliers are detected, highlighting potential anomalous operating conditions in specific WTGs. For these identified WTGs, the Shapley coefficients are computed using the reference wind turbine model. The obtained rankings for these target wind turbines are then compared with that of the reference WTG to identify any disparities. Variables exhibiting divergent ranking positions serve as alarm signals for wind farm operators, aiding in WTG condition monitoring. This approach enables the timely detection and investigation of deviations from expected performance, ensuring that prompt corrective actions can be taken to maintain the optimal functioning of the wind farm.

The methodology is here discussed in detail, highlighting its key components, before introducing the workflow of the XAI-based methods. Mathematically, the process above leads to the development of a model:

(1) y←f(X)

where $y\in {ℜ}^{N\times 1}$ is the power output vector of *N* samples, and $X\in {ℜ}^{N\times M}$ is the multivariate input matrix with *M* variables (or features). The latter includes the variables selected by SFS from a set of P≥M predictors $X=\{x_{1},\cdots ,x_{P}\}$, and validated by Shapley coefficients. For the generic *n*th sample, Equation (1) is y[n]←f(X[n,]).

The XAI workflow employed in this work exploits the map of Equation (1) in several ways (as is visible from the flow diagram in Figure 1) and optimizes such map using a loss function (i.e., an accuracy metric). Therefore, before describing in detail the XAI methods in Section 2.3, we describe in Section 2.1 the particular map y←f(X) selected in this work (GPR) and we introduce in Section 2.2 the employed accuracy metrics, indicating at which step of the workflow they are employed.

### 2.1. Gaussian Process Regression

Gaussian regression is the nonparametric kernel-based probabilistic model used in this study to build the multivariate wind power curve according to the map y←X.

Particularly, the principles of Gaussian process regression are extensively explained in [33], and we refer to their work for detailed information, as well as to the work in [12,34] for wind turbines SCADA applications.

Mathematically, a Gaussian process is a stochastic process defined in terms of mean m(x) and its covariance $k(x,x^{\prime })$ function, as in Equation (2):

(2) $$f\left(x\right)≃GP\left(m\left(x\right),k\left(x,x^{\prime }\right)\right)$$

where *m* is the expected value (Equation (3)):

(3) $$m\left(x\right)=E\left[f(x)\right]$$

and the covariance is defined in terms of its associate probability density function (Equation (4)):

(4) $$p\left(x,m,K\right)=\frac{1}{{\left(2\pi \right)}^{\frac{q}{2}}{\left|K\right|}^{1/2}}e^{-\frac{1}{2}{\left(x-m\right)}^{T}K^{-1}\left(x-m\right)},$$

where *q* is the number of input variables (which means the dimension of the vector x) and $\left|K\right|$ is the determinant of the covariance matrix, whose order is equal to the number of available samples in the training set ($n_{tr}$). The terms x and $x^{\prime }$ indicate two points of the space, which correspond to two rows of matrix X. The mean m(x) can be assumed without loss of generality to be zero. The meaning of an element $K(x,x^{\prime })$ is a measure of how different points *x* and $x^{\prime }$ are.

The kernel covariance function selected in this work is the squared exponential, which is given in Equation (5):

(5) $$k_{SE}\left(x,x^{\prime }\right)=\sigma_{f}^{2}e^{-\frac{\left|\right|{\left(x-x^{\prime }\right)}^{2}\left|\right|}{2l^{2}}}+\sigma_{n}^{2}\delta \left(x,x^{\prime }\right),$$

where $\sigma_{f}^{2}$ (variance), $l^{2}$ (length scale), and $\sigma_{n}^{2}$ (noise variance) are the model hyperparameters. In this study, the hyperparameters $\sigma_{f}^{2}$, $l^{2}$, and $\sigma_{n}^{2}$ are estimated using the Bayesian optimization. A detailed analysis of the covariance function selection for this kind of application is provided in [34]. The elements of K are, therefore, the variances of each input variable in the diagonal and the covariance (computed according to Equation (5)) between pairs of input variables in the off-diagonal. Each element of matrix K is computed by applying Equation (5).

The assumption of the Gaussian process is, therefore, that the vector output y to predict is given by Equation (6):

(6) y=f(X)+ϵI,

where f is the sample GP function, and ϵ is the white noise and I the identity matrix. The model training is based on the log-likelihood maximization of the probability (Equation (4)) of observing the training data set output $y_{tr}$ given the input observations matrix $X_{tr}$, as in Equation (7):

(7) $$log\left(p\left(y_{trn}|X_{trn}\right)\right)=\frac{1}{2}y_{trn}^{T}K^{-1}y_{trn}-\frac{1}{2}log\left(\left|K\right|\right)-\frac{1}{2}q\log\left(2\pi \right).$$

where $X_{trn}$ and $y_{trn}$ are the training input and output data, respectively.

The trained model can be used to predict the test output given a new set of unseen data $\left(X_{test},y_{test}\right)$ considering that $y_{test}$ and $f(X_{test})$ follow a joint normal distribution, as shown in Equation (8):

(8) $$\left[\begin{array}{c} y_{trn}\\ f(X_{test}) \end{array}\right]∼N\left(0,\left[\begin{array}{cc} K\left(X_{trn},X_{trn}\right)+\sigma^{2}I & K(X_{trn},X_{test})\\ K(X_{test},X_{trn}) & K(X_{trn},X_{trn}) \end{array}\right]\right)$$

Particularly, $f(X_{test})$ is the estimated function, whose weights are returned by using Gaussian likelihood and computing the posterior distribution of the weights with respect to the test data [35]. For this kind of model, the conditional distribution $p(f\left(X_{test}\right)|X_{trn},y_{trn},X_{test})$ is a multivariate normal distribution, with mean and variance given in Equations (9) and (10):

(9) $$K\left(X_{test},X_{trn}\right){\left[K\left(X_{trn},X_{trn}\right)+\sigma^{2}I\right]}^{-1}y_{trn}$$

(10) $$K\left(X_{test},X_{test}\right)-K\left(X_{test},X_{trn}\right){\left[K_{trn}\left(X_{trn},X_{trn}\right)+\sigma^{2}I\right]}^{-1}K\left(X_{trn},X_{test}\right)$$

where $K(X_{test},X_{test})$ is the covariance matrix of the test data, whose size is $\left[N_{test}\times N_{test}\right]$; and $K(X_{trn},X_{test})$ ($K(X_{test},X_{trn})$) is the covariance matrix between training/test and test/ training data, whose size is $\left[N_{trn}\times N_{test}\right]$ ($\left[N_{test}\times N_{trn}\right]$). Equation (9) returns the corresponding vector of the estimated target ${\hat{y}}_{test}$, where the single element is returned by considering the single point of $X_{test}$.

### 2.2. Accuracy Metrics

The proposed methodologies utilize an iterative approach, where the accuracy of the model is monitored using defined loss functions in block (A) of Figure 1. The following metrics are therefore used to assess the model’s performance. Let R[i] be the *i*-th residual, defined in Equation (11) as the difference between the *i*-th measurement and the estimate provided by the model when the *i*-th row of the X is fed as input:

(11) R[i]=y[i]−f(X[i,]),

where y[i] and f(X[i,]) are the true and estimated value, respectively.

The root-mean-square error (RMSE) is defined as in Equation (12):

(12) $$\mathrm{RMSE}=\sqrt{\frac{\sum_{i}^{N_{test}}{\left(R\left[i\right]-\bar{R}\right)}^{2}}{N_{test}}},$$

where $\bar{R}$ is the average residual and $N_{test}$, is the number of samples in the evaluation data set. The mean absolute error (MAE) is defined in Equation (13):

(13) $$\mathrm{MAE}=\frac{1}{N}\sum_{i}^{N_{test}}\left|R[i]\right|.$$

Furthermore, the 95th percentile of the vector composed of the absolute value of the residuals is selected as a meaningful accuracy metric (indicated as $\left|R_{95\%}\right|$). The rationale for this is that hopefully, the constructed method should give high prediction errors with an extremely low probability. The 95th percentile is computed using the T-digest approximation [36]. These metrics are normalized to the rated power, making them comparable with the literature.

The metrics are employed as follows:

- The RMSE is used as a loss function at each step of the sequential feature selection process, block (A) of Figure 1;
- The RMSE, the MAE, and $\left|R_{95\%}\right|$) are used to evaluate the accuracy of the GPR model trained with the selected features, in relation to the state of the art in the literature. This is performed in block (A) of Figure 1. For completeness, a comparison is set up against a univariate method, taking as input solely the wind speed, and the same accuracy metrics are employed.
- The RMSE of the GPR model trained with the reference wind turbine and employed on the data of the target wind turbines is employed to identify outlier wind turbines that are suspected of anomaly. This is performed in block (B) of Figure 1.

### 2.3. XAI Methods

The model described in Section 2.1 can be executed with any selection of input variables, and its accuracy can be evaluated using the metrics in Section 2.2. The two XAI methods proposed in this paper aim to provide insights into the importance of each possible input variable. The SFS method starts with an empty set of input variables. It adds them one at a time, identifying at each round of the GPR regression the variable that reduces a certain loss function the most (in this work, root-mean-square error). On the other hand, the calculation of the Shapley coefficients works in the opposite direction. It begins with the set of covariates selected at the output of the SFS algorithm and then computes the difference in model output for input data with or without each feature by marginalizing over subsets that do not contain the feature. The former method ranks input variables based on how much they reduce errors, while the latter ranks them based on how much they explain the error. These methods can be seen as two complementary ways of explaining feature importance.

#### 2.3.1. Sequential Feature Selection

The goal of SFS is to identify the input variables that, when added, lead to an improvement in prediction accuracy. This algorithm is crucial in transitioning from relying solely on expert knowledge to having a clear criterion for selecting the input variables for a power curve model.

The SFS algorithm, applied in block (A) of the workflow of Figure 1, follows the steps outlined below:

1. Initialize a matrix with null dimensions $X_{M=0}$ to store the most significant predictors from the set $X_{M=0}=\left\{x_{1},\cdots ,x_{j},\cdots ,x_{P}\right\}$, with *P* input variables, a vector of output y, a counter variable *M* set to 1, and ${\mathrm{RMSE}}_{M=0}=\infty$. *P* represents the number of variables present in the original data sets, where each variable can be considered as a column vector with *N* samples. As more features related to the operating state of wind turbines are included from scratch, the value of *P* increases accordingly, but for a certain data set, *P* is fixed, and is therefore not a parameter of the model.
2. Repeat until ${\mathrm{RMSE}}_{M}>{\mathrm{RMSE}}_{M-1}$
  (a) Iterate for each *j*-th variable $x_{j}$, where $j\in [1,|X_{M-1}|]$
    - Split the samples of set $X_{M-1}$ into training and validation sets using *K*-fold cross-validation.
    - For each k∈[1,K], build a GP-based model using the *k*-th training set, and merge the variables of $X_{M-1}$ with the *j*-th variable $x_{j}$ in the set $X_{M-1}$. Test each *j*-th model on each one of the K−1 validation sets.
    - Compute the average out-of-sample ${\mathrm{RMSE}}_{M}$ (loss function) across the K sample-sets for each *j*-th model.
    - Sort the $|X_{M-1}|$ models and select the variable from $X_{M-1}$ that corresponds to the model with the lowest ${\mathrm{RMSE}}_{M}$.
  (b) Remove the selected variable from $X_{M-1}$ and merge by column with the matrix $X_{M-1}$, which contains the variables previously selected, to create $X_{M}$.
  (c) Increase the counter *M* of one unit.
3. End the algorithm when the loss function stops decreasing, and the matrix $X=X_{M}$ contains the most informative *M* input variables.

#### 2.3.2. Shapley Coefficients

The application of Shapley coefficients in machine learning applications is derived from coalition games, where it is interested in assigning the player a payout by assessing its contribution to reaching the total amount [31]. In this specific case, the aim is to evaluate the role of each feature in the prediction’s quality after constructing the set of best predictors X using the SFS algorithm. Thus, to understand the reasoning behind computing the Shapley coefficients, it is useful to start from the model built in Equation (1) for the *n*-th instance, which is simplified to a linear model for this explanation:

(14) y[n]=X[n,]β[n]

The notation in Equation (14) indicates the product between the *n*-th row of X and the column vector of β coefficients. Equation (14) can be compactly written as Equation (15):

(15) y=Xβ.

In this case, the *j*-th Shapley coefficient can be computed as follows in (16):

(16) $$\phi_{j}=\beta_{j}x_{j}-\beta_{j}E\left(X\right),$$

which represents the difference between the effect of the *j*-th feature and the average effect (calculated as the expectation value *E*). The coefficient $\phi_{j}$ explains how much the average error depends on the *j*-th feature, and thus ranks the features.

The above concept can be generalized for nonlinear models, as performed, for example, in [32]. If an output is dependent on *M* features, the coefficient given can be defined as in Equation (17):

(17) $$\Phi_{j}=\sum_{S\subseteq \{1,\cdots ,M\}\setminus \{j\}}\frac{|S|!\left(M-|S|-1\right)}{M!}\cdot \left(g\left(S\cup \{j\}\right)-g\left(S\right)\right)$$

where *S* is a subset of the *M* features extracted by the SFS from the set X and stored in the matrix X, with the *j*-th feature excluded. The functions g(S∪{j}) and g(S) are the expected values of the prediction, with all features and with the exclusion of the *j*-the feature, respectively. The functions $g_{x}\left(S\right)=g\left(S\cup \{j\}\right)-g\left(S\right)$ are estimated based on the observed input distribution, as in Equation (18). It can be interpreted as the prediction for the feature values in a specific set S while marginalizing over the features that are not included in set S.

(18) $$g_{x}\left(S\right)=\int f\left(x_{1},\cdots ,x_{M}\right)dP_{X\notin S}-E_{X}\left(f\left(X\right)\right)$$

This formulation, which is the one employed in this work, is called the random baseline Shapley [32]. The feature with the highest Shapley coefficient is considered the most important, as it explains the highest proportion of the residual between the measurement and model prediction. Due to the high computational demands (a sum with $2^{M}$ terms should be considered for each coefficient), it is suggested to compute the Shapley coefficients on a representative subset of the data. In this work, 10% of the data are selected. The computational cost is in the order of 24 h for each set of Shapley coefficients, using a laptop with 3.1 GHz Intel Core and Matlab 2023. In this study, the coefficients are reported as average absolute values, as the mean prediction error over a set is expected to be zero, and using the absolute value helps emphasize the feature importance. These coefficients are computed considering the *M* extracted features according to the SFS algorithm.

### 2.4. Application for Condition Monitoring

Once a workflow like the one described above has been set up, it can be employed in various ways for condition monitoring. The practical implementation depends on the specific test case and the characteristics of the data set. In general, a space–time approach has been discussed in [16] as helping to monitor wind turbine fleets. This means that a data-driven model can be utilized to analyze the time evolution of a single system or to compare nearby wind turbines installed at the same site, or both. The former approach is more suitable for tracking the evolution of an incoming fault, while the latter is useful for highlighting different operating modes and/or systematic errors between wind turbines that are expected to be equal but may not be in practice.

In this study, the latter approach is advocated and implemented in blocks (B) and (C) of the workflow (Figure 1). The detailed steps are the following:

- The multivariate model, trained with the data from the reference wind turbine, is utilized to simulate the output for all the wind turbines in the farm based on the given input variables.
- Error metrics (Equations (12) and (13)) are computed for all the wind turbines in the farm to identify outliers.
- For the selected outlier wind turbines, the Shapley coefficients are computed using the workflow described in Section 2.3.2.
- The difference between these coefficients and the corresponding values for the reference wind turbine is used as a target for identifying the component suspected of anomaly.
- The 3σ rule is applied, where the average Shapley coefficients for the target wind turbine are compared to those of the reference wind turbine in terms of standard deviations (σ). Covariates deviating more than 3σ are identified through this procedure.

It should be noted that unlike standard IEC-like power curve models, it is not expected that the error metrics would be of similar magnitude when simulating a different wind turbine from the one used for training. This is because the relationship between the operational parameters and the power output depends on the health status of the system, which can vary among different wind turbines, even within the same plant. However, an outlier in the space–time comparison should still be distinguishable, as supported by the discussion in Section 3.

The proposed application for condition monitoring represents an innovation with respect to the state of the art, thanks to the distinctive features of explainability. Actually, the vast majority of the approaches to wind turbine condition monitoring through SCADA data analysis implicitly assume a form of supervision, which is the selection of the target to monitor; for example, the temperature measured at meaningful rotating components of the system. The method formulated in this work is completely unsupervised, thanks to explainability. Starting from a set of features, as vast as possible, the differences in the operational behavior between reference and target wind turbines are identified. This approach can benefit the wind energy community in individuating anomalies that are substantially hidden, and thus overlooked.

## 3. Case Study

### 3.1. Test Cases and Data Sets

The two test cases of this work were selected because they are representative examples of the most employed technologies worldwide. The wind turbines are pitch-controlled, and consequently have variable rotational speeds. The substantial difference between the test cases reported in Table 1 is that the former has hydraulic blade pitch actuation, while for the latter case, the blade pitch control is electrical. The advantages and drawbacks of both types of blade pitch control are a topic attracting attention in the literature [37].

A healthy reference wind turbine is manually selected for each test case based on scrutiny of the maintenance reports and alarm logs. The set of possible input variables for Test Cases 1 and 2 are reported in Table 2 and Table 3, respectively.

The starting set of variables reported in Table 2 and Table 3 are selected based on expert knowledge. The rationale behind the selection is to include wind speed measurements at first, as they are generally considered the variable that explains most of the variance in power output [20]. Additionally, variables related to wind turbine operation and control (rotational speed and blade pitch), mechanical response (tower vibrations for Case 1, drivetrain vibrations for Case 2), and internal temperatures are included as meaningful indicators. Furthermore, variables specific to the blade pitch control system are also included, such as pressures and cylinder traveled distance for Case 1, and blade pitch motor currents for Case 2.

A few differences are worth noting for appreciating the peculiarities of the test cases as regards the wind speed measurement, which is known to be a critical point [38,39]. In Test Case 1, more recent wind turbines are used, which are controlled by AI-based algorithms developed by the manufacturer. The wind speed measurement reported in Table 2 is an artificial signal estimated by the controller using two physical sensors and operational variables of the wind turbine, and hence, it is included as a possible input variable in Table 2. On the other hand, in Test Case 2, the wind turbines are less recent, and the wind speed estimated by the controller is simply the average of the two measurements collected by the physical sensors. Therefore, only the physical sensors’ measurements are reported in Table 3 for Test Case 2. The presence of two sensors is interesting, as the difference between them is likely related to the yaw angle and the extracted power [40].

### 3.2. Data Preprocessing

The data for both test cases are preprocessed using the following steps:

- Data corresponding to wind turbines running in normal operations are kept, while other operating modes are discarded.
- Data below the rated speed (13 m/s) are retained, as power curve modeling above the rated speed is considered trivial.
- Wind power curtailment operation of industrial wind turbines, which may occur due to grid or noise reduction requirements, is filtered out from the normal behavior model of wind turbine power. This is performed by identifying outliers based on blade pitch deviation from the average curve as a function of the wind speed, with deviations greater than $2.5^{\circ }$ degrees leading to rejection of the measurement. Advanced data clustering methods can also be employed for this purpose [41].
- The output of each input and output variable is standardized by subtracting its mean and scaling by its standard deviation.

### 3.3. Experiments Settings

The SFS algorithm is run on a random 10% subset of the training data set to reduce its high computational costs.

A total of 30 random values for the hyperparameters are tested, and the model’s performance is evaluated using 10-fold cross-validation to identify the hyperparameter values that yield the minimum average error metrics in the development of a GP regression-based multivariate model. The values are reported in Table 4.

A univariate benchmark based on the same regression type is also considered for comparison with the state of the art. Half of the data are used for training, and half are used for evaluating the accuracy of the selected model.

## 4. Experimental Results

A comprehensive set of experiments is conducted in this study, taking into account the distinct behavior of wind turbines equipped with different pitch actuators. Two separate data sets, corresponding to Test Cases 1 and 2 outlined in Section 3, are considered, and the main results are summarized accordingly.

### 4.1. Test Case 1

#### 4.1.1. Discussion of the Input Variables

**Figure 2:**
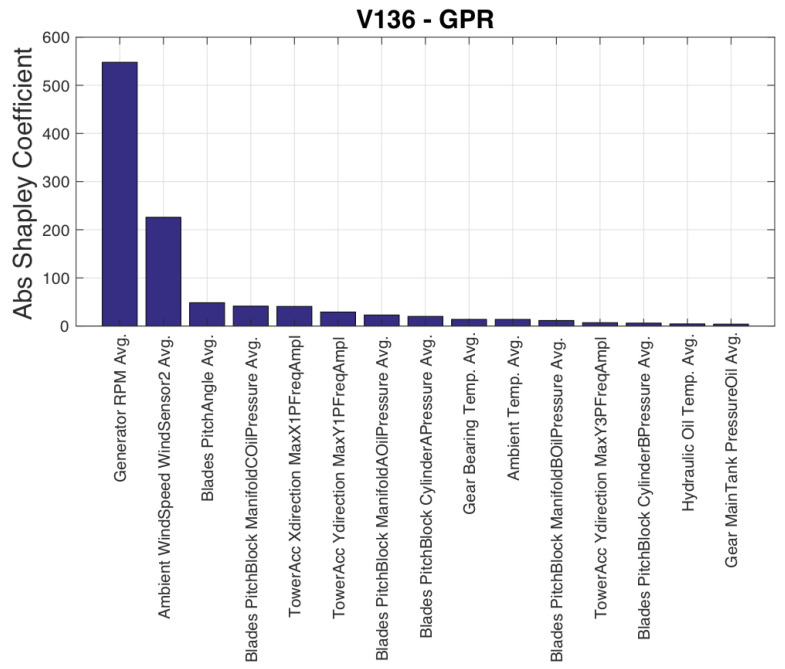
Input variables ordered by their Shapley coefficient, Test Case 1.

The sequence of selected input variables is reported with their selection order in Table 5. The most important result is that the selection includes a large number of variables (15), which prompts a critical review of the existing literature. Rotational speed and blade pitch are considered important covariates in addition to wind speed [12]. Table 5 indicates that SCADA-collected data sets contain variables related to blade pitch behavior, such as pressures of the blade pitch block, which are more appropriate than the blade pitch itself for modeling output power. However, these variables have been overlooked in the literature, making this work a stimulus for deeper utilization of SCADA data sets. It is worth noting that ambient temperature is considered an important factor in data-driven power curve models, as mentioned in [42], yet Table 5 shows that it is selected only as the thirteenth variable. Figure 2 reveals that wind speed and rotational speed are the most important variables for an accurate power model according to their Shapley values, as they have the highest explanatory power and account for the highest portion of the average error.

#### 4.1.2. Model Assessment

**Figure 3:**
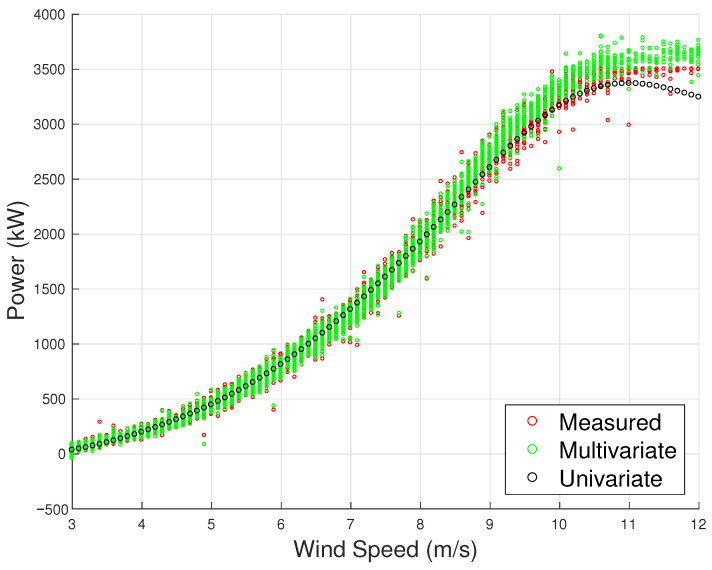
The measured power curve and the estimated one through the multivariate and univariate model, Test Case 1.

In order to appreciate the rationale for employing a multivariate model for power, the accuracy metrics of the model using the covariates from Table 5 are compared to those of a univariate model that only takes wind speed as input. As shown in Table 6, the proposed multivariate model outperforms the state of the art in the literature [12,27], which is notable considering the challenge of modeling power for wind turbines with large rotors. Furthermore, the error metrics are considerably higher for the univariate case, with the MAE almost double compared to the multivariate case. The 95th percentile of the absolute difference between model estimates and measurements reaches 5.25% in the univariate case, which means that there is a non-negligible probability of predicting the power with a high error. This quantity considerably diminishes for the multivariate method proposed in this work. These findings are also reflected in Figure 3, where the univariate model fails to reproduce the real-world dispersion of the observed curve, while the multivariate model performs significantly better.

#### 4.1.3. Application for Condition Monitoring

**Figure 4:**
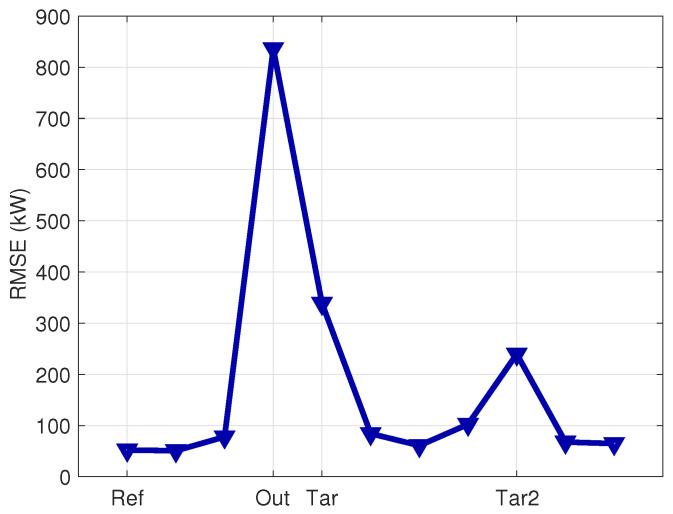
RMSE for the evaluation of the model trained with the data of the Ref wind turbine on all the wind farm, Test Case 1. The Ref and selected Tar and Tar2 wind turbines are indicated, as well as the outlier one.

When simulating the output for the other 10 wind turbines in the farm using the model trained with data from the reference wind turbine as in block (B) of Figure 1, the average accuracy metrics generally worsen. For 8 out of 10 wind turbines, the obtained RMSE ranges from 0.97 to 1.97 times the RMSE of the reference wind turbine. However, for three wind turbines, the obtained RMSE is remarkably higher than the reference. One wind turbine, which has an RMSE 16 times that of the reference, is identified to be affected by an anemometer fault, which is considered a data quality issue, and is discarded from further analysis. Two wind turbines, labeled *Tar* and *Tar2*, are selected as targets because their RMSE is, respectively, 4.6 and 6.5 times that of the reference, as can be seen from Figure 4. There is no evident underperformance issue associated with these wind turbines.

**Figure 5:**
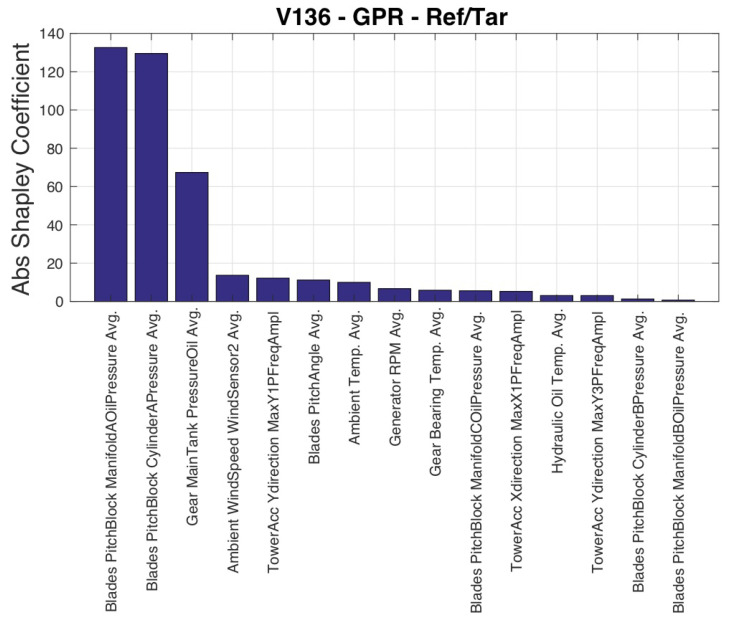
Input variables ordered by their difference in the Shapley coefficient between reference and target, Test Case 1.

**Figure 6:**
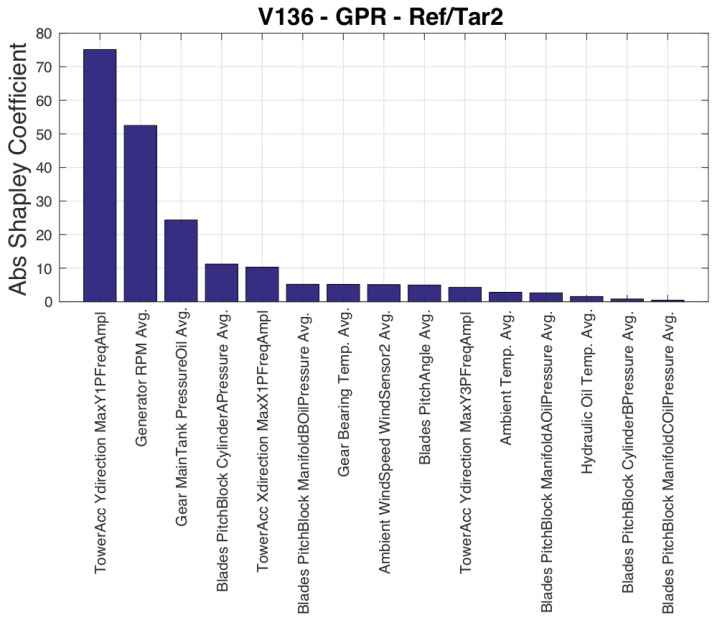
Input variables ordered by their difference in the Shapley coefficient between reference and target 2, Test Case 1.

The procedure described in Section 2.4 and block (C) of Figure 1 is applied, and Shapley coefficients are computed using the reference model on the data of the target wind turbines. In order to highlight the differences compared to the reference, the Shapley coefficients for the target wind turbines are subtracted from the reference Shapley coefficients. The results are shown in Figure 5 and Figure 6, from which it is evident that one target wind turbine has an anomaly related to hydraulic pitch at one blade (A), and another target wind turbine is affected by anomalous transverse tower vibrations, likely related to blade pitch imbalance.

**Figure 7:**
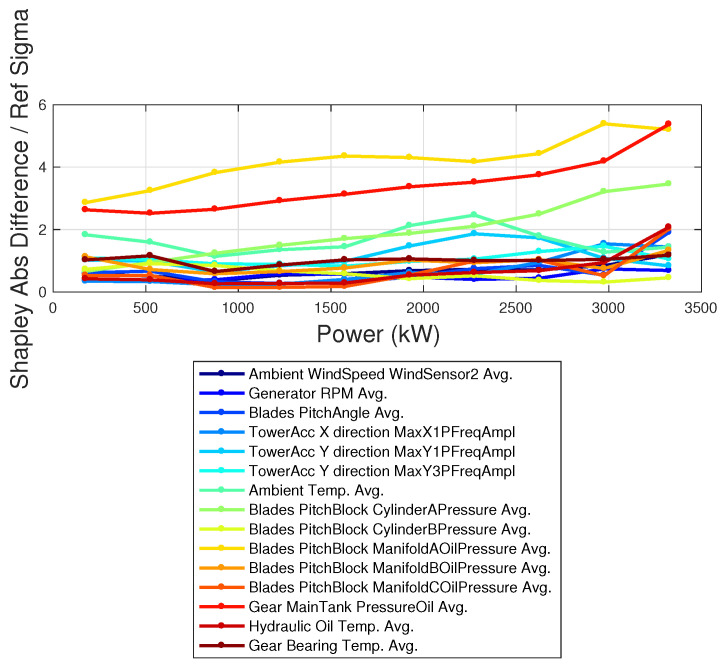
Absolute difference of the Shapley coefficients between target and reference, in units of the standard deviation. The set of Shapley coefficients is averaged per power intervals. Test Case 1.

**Figure 8:**
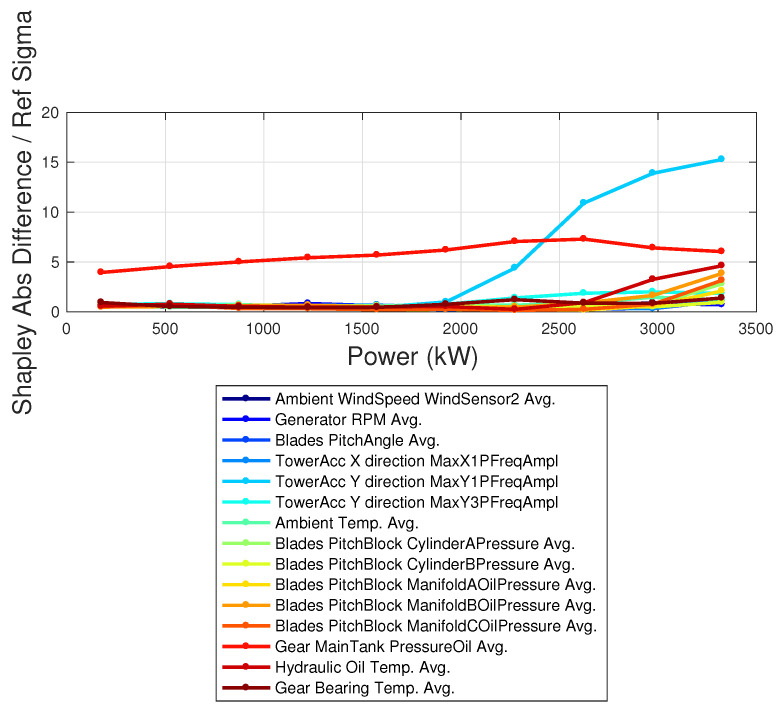
Absolute difference of the Shapley coefficients between target 2 and reference, in units of the standard deviation. The set of Shapley coefficients is averaged per power intervals. Test Case 1.

In Figure 7 and Figure 8, the relative Shapley coefficients (target with respect to reference) are reported in units of standard deviation for each measurement channel and averaged per power intervals of the target wind turbines. The rationale for such analysis is identifying the subcomponents related to hidden operation anomalies, as well as the most affected power regimes. From Figure 7, it arises that the pitch of blade A is anomalous, and reasonably, the anomaly grows with the power, i.e., with the load. A considerable alarm is also raised for the gear main tank pressure. For the wind turbine Tar 2 (Figure 8), the anomaly regards the gear main tank pressure, and especially the transverse tower vibrations, for high power (above 2 MW). Notably, the generator speed is not found to be significantly anomalous for wind turbine *Tar2* according to the 3σ rule.

These results support the usefulness of the proposed method in identifying anomalies even before they become severe enough to visibly affect wind turbine performance. The advantage with respect to the state of the art is the lower level of supervision of the algorithm, which is compensated by the explainability. In other words, most state-of-the-art methods require knowing a priori what target should be monitored, i.e., where the anomaly should be located. The method proposed in this work does not require such prior knowledge, and is therefore useful for individuating anomalies not likely identified by the state-of-the-art methods. The health status of the hydraulic blade pitch system is identified as a critical aspect that should be carefully monitored, as recent studies have shown that it is likely related to performance degradation with age [43]. As an aside, it should be noticed that the present analysis has been integrated in the O&M of the wind farm owner, and the anomalies individuated through the proposed method are being analyzed and corrected by the manufacturer.

### 4.2. Test Case 2

#### 4.2.1. Discussion of the Input Variables

**Figure 9:**
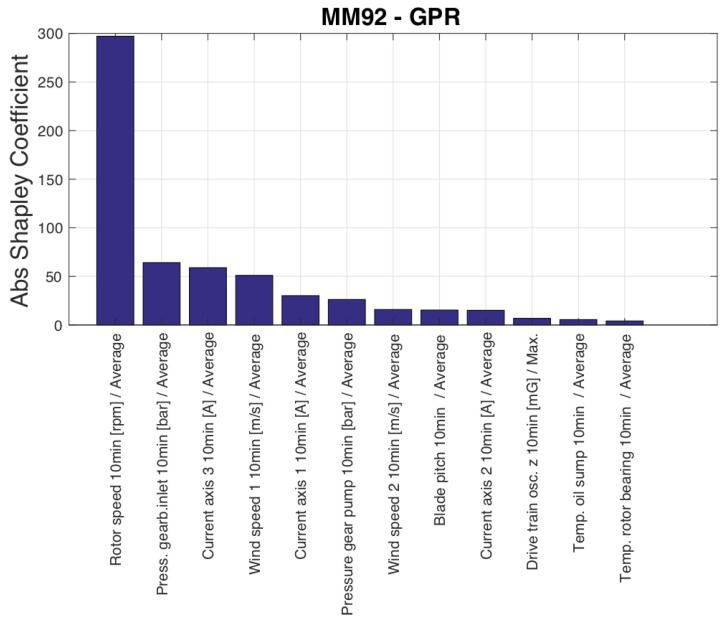
Input variables ordered by their Shapley coefficient, Test Case 2.

The input variables selected by the SFS are reported in Table 7. The conclusions are qualitatively similar to Test Case 1, with blade pitch being selected as the ninth variable, and a variable related to blade pitch control (pitch motor current) being selected even before rotational speed. Figure 9 qualitatively resembles Figure 2, indicating that the behavior of the rotational speed is a significant factor in the discrepancy between estimated and measured power. This suggests that efforts to improve the data-driven modeling of wind turbine power should focus on accurately reproducing the rotational speed behavior.

#### 4.2.2. Model Assessment

**Figure 10:**
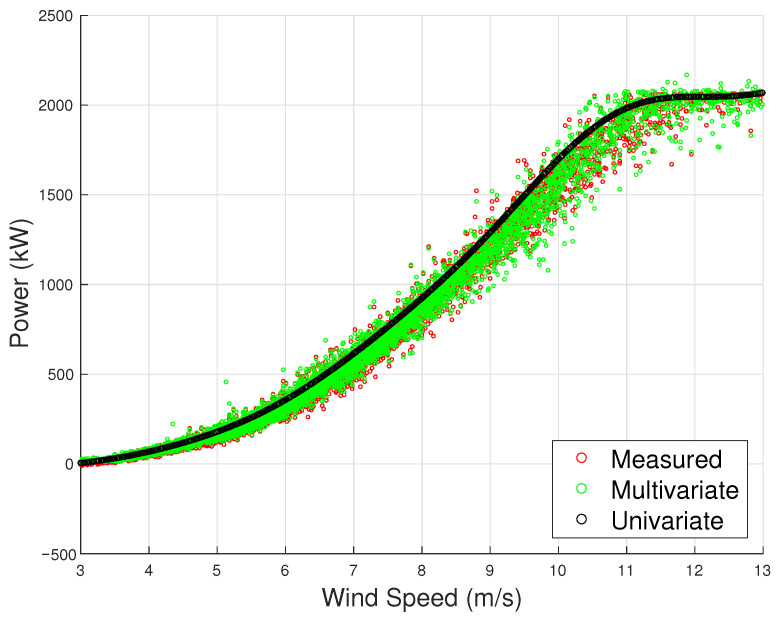
The measured power curve and the estimated one through the multivariate and univariate model, Test Case 2.

The accuracy metrics for the selected model are reported in Table 8 and are compared against a univariate model trained and tested on the same period. Similar to the first test case, the multivariate model outperforms the state-of-the-art literature model, supporting the use of a large set of input variables. The univariate model has an average MAE more than double that of the multivariate model. In particular, the comparison of the $\left|R_{95\%}\right|$ metric highlights that the use of the multivariate method remarkably decreases the highest error, which is achieved with a confidence interval of 95%. The ability of the multivariate model to accurately reproduce the real-world dispersion of the data, unlike the univariate model, can be clearly observed in Figure 10.

#### 4.2.3. Application for Condition Monitoring

**Figure 11:**
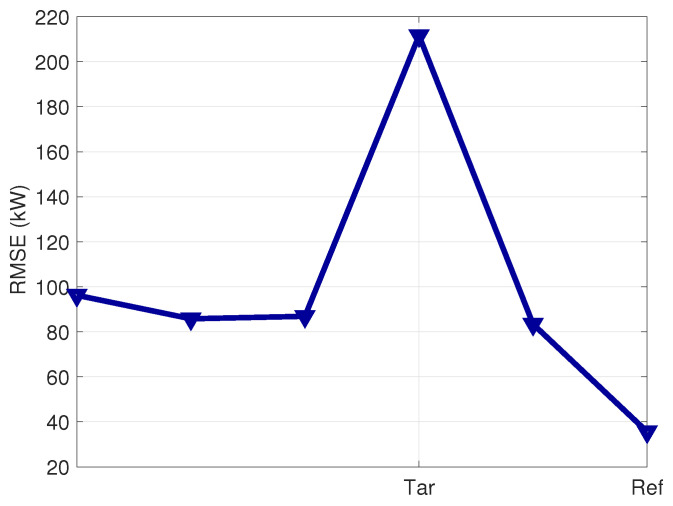
RMSE for the evaluation of the model trained with the data of the Ref wind turbine on all the wind farm, Test Case 2. The Ref and selected Tar wind turbines are indicated.

**Figure 12:**
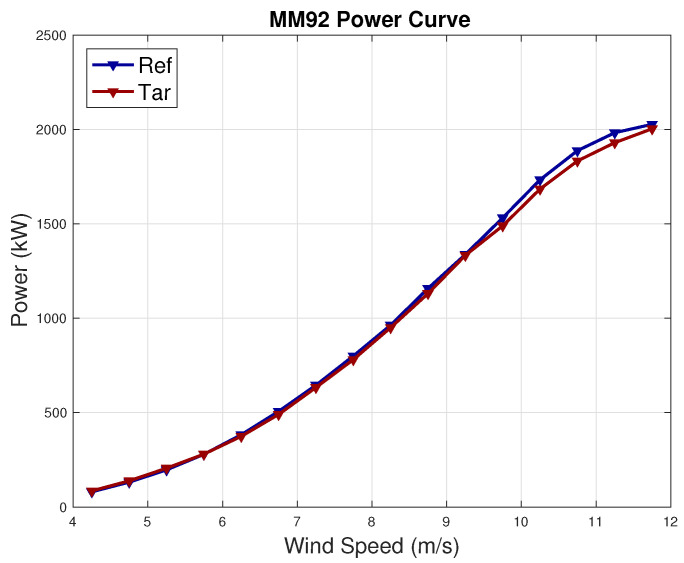
Average power curve of the reference and target wind turbine in Test Case 2.

When simulating the output on the other five wind turbines in the farm by using the model trained with the data of the reference wind turbine (as indicated in block (B) of Figure 1), the average error metrics increase. For 4 wind turbines out of 5, the obtained RMSE ranges from 2.4 to 2.7 times the RMSE of the reference wind turbine. For 1 wind turbine, selected as the target for further inspections, the achieved RMSE is 6 times the RMSE of the reference wind turbine. This can be visualized in Figure 11. Therefore, such a wind turbine is selected as the target, and the workflow indicated in Section 2.4 and block (C) of Figure 1 is set up. Prior to that, the average power curve for the reference and target wind turbines are compared through a visual inspection in Figure 12, from which it arises that the target wind turbine is slightly underperforming.

**Figure 13:**
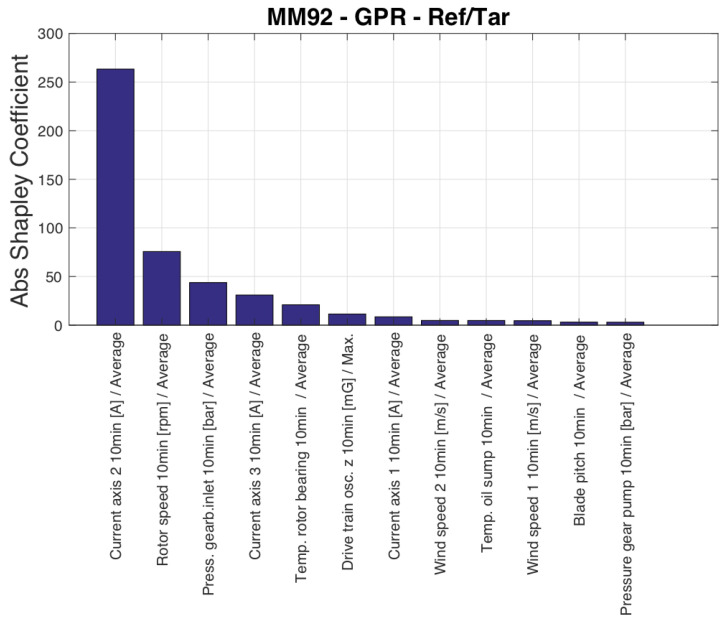
Input variables ordered by their difference in the Shapley coefficient between reference and target, Test Case 2.

**Figure 14:**
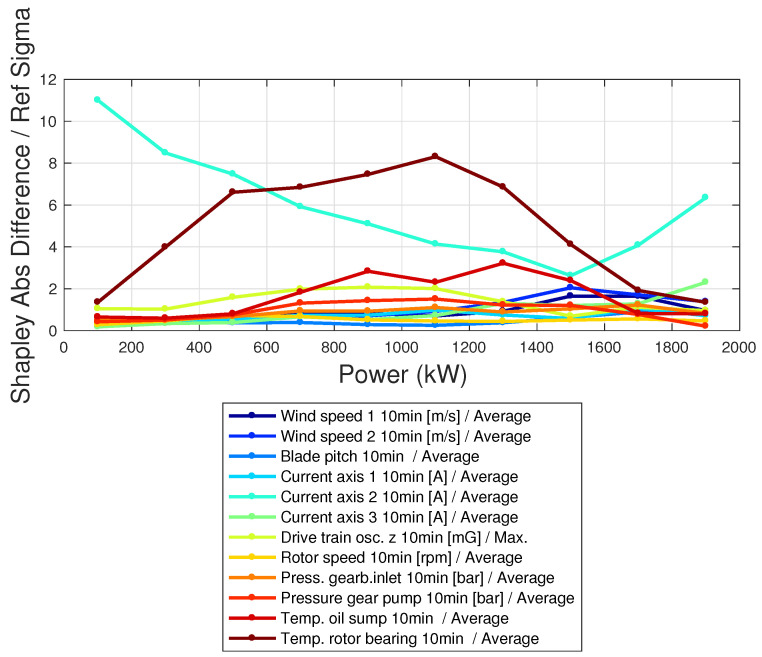
Absolute difference of the Shapley coefficients between target and reference, in units of the standard deviation. The set of Shapley coefficients is averaged per power intervals.

The Shapley coefficients are computed using the data of the target wind turbine with the model trained on the reference wind turbine. The difference between the average Shapley coefficients of the target and reference wind turbines is shown in Figure 13. In Figure 14, the absolute difference between the Shapley coefficients of the reference and target wind turbines is reported, in units of the standard deviation, as a function of the power of the target wind turbine. From Figure 14, it follows that an anomaly above 3σ occurs for the pitch motor of blade 2 in the target wind turbine and for the temperature of the rotor bearing. Furthermore, Figure 14 highlights that the anomaly at the blade pitch motor is especially high for low powers and for high powers, which are the regimes where the blade pitch works most. The anomaly at the rotor bearing temperature instead is most remarkable in the regime of variable rotational speed, which is reasonable because of more rotational speed and more heat. The application of XAI techniques to a multivariate data-driven model with multiple input variables therefore provides a novel insight for understanding the reasons behind the observed underperformance and for identifying anomalies that would be invisible through naive scrutiny.

## 5. Discussion

The results collected in Section 4 respond to two challenging issues related to wind turbine systems monitoring:

- Selecting what features are most representative for describing a multivariate normal behavior of a system as complex as a wind turbine;
- Highlighting anomalies in the system operation behavior, which would not be visible through state-of-the-art scrutiny, and ascribing it to certain subcomponents.

The answer that this work provides to the above two issues is related to explainability. Regarding the feature selection, the criterion adopted in this work is minimizing the loss function in modeling the power of the wind turbine as a function of multiple input variables. This is due to the fact that ultimately, the interest is in attaining maximum efficiency, which requires monitoring the produced power as accurately as possible. The results collected in Section 4.1.2 and Section 4.2.2 indicate that the formulated models are substantially more accurate than benchmark models in the literature, and considerably diminish the probability of predicting the expected power with a high error. Furthermore, the analysis contributes in general to the insight into the use of SCADA-collected measurements for wind turbine normal behavior modeling. The results of this work indicate that the wind turbine SCADA systems collect dozens of measurement channels, which should be included in the monitoring algorithm differently with respect to the state of the art in the literature.

Regarding the condition monitoring application, the contribution provided by this work is the possibility of assuming a lower level of supervision with respect to the state of the art, which is compensated by the features of the XAI algorithm. In other words, the state of the art is typically based on the a priori identification of the target measurement channels to monitor, for which a normal behavior model is constructed. The workflow structured in this work instead starts from a vast set of features, and through the comparison between reference and target wind turbines, identifies the features and the machine working regime, which are likely anomalous. The added value of the XAI algorithm consists, therefore, in highlighting anomalies that are not visible through state-of-the-art scrutiny. Indeed, for the test case of interest, these anomalies were not highlighted by standard methods, and the indications coming from this work were useful for planning the maintenance.

## 6. Conclusions

The present work focuses on the use of XAI techniques for developing multivariate wind turbine power curve models and applying them for condition monitoring in two real-world test cases with different blade pitch control technologies (hydraulic vs. electrical).

The main conclusion drawn from this study is that the use of multivariate models for monitoring wind turbine power is highly recommended. With an appropriately structured workflow, as demonstrated in this work, multivariate models offer significant advantages in terms of accuracy and explainability compared to the state of the art in the literature. The improved accuracy is achieved by using a broader set of covariates and selecting the most relevant ones through sequential feature selection. The achieved model, despite being a black box (e.g., GPR), remains explainable through the use of Shapley coefficients to assess the contribution of each selected covariate to the average error metrics.

Particularly, the experiments in this manuscript highlight the following: (i) The role of the blade pitch as an input variable for wind turbine power modeling should be critically re-evaluated. Variables related to hydraulic (e.g., actuator pressures) or electrical (e.g., motor currents) control of the blade pitch may be more suitable for accurate power modeling; (ii) The potential of condition monitoring applications based on multivariate data-driven models and space–time comparison between nearby wind turbines is promising. In the selected test cases, anomalies related to blade pitch control and tower vibrations were identified in three wind turbines. These anomalies were not highlighted by state-of-the-art methods, which (differently with respect to the method of this work) typically require an a priori identification of the target where the anomaly is expected to occur.

There are several potential future directions of this work. In addition to the space comparison pursued in this study, a deeper analysis could involve monitoring the temporal evolution of the Shapley coefficients for each wind turbine to detect changes related to incoming faults [44,45]. Furthermore, a multi-input–multioutput model based on similar principles as in this work could have wider applications in the field of wind turbine system condition monitoring, as demonstrated in recent studies [46].

## Figures and Tables

**Table 1:** Main features of the test cases.

	N. Tur.	Rated Power (MW)	Rotor Diameter (m.)	Blade Pitch	Data Set
Case 1	11	3.4	136	Hydraulic	1 August–9 October 2022
Case 2	6	2	92	Electrical	1 August 2020–10 January 2021

**Table 2:** List of the possible input variables for Test Case 1.

Wind Speed (m/s)
Wind Speed Sensor 1 (m/s)
Wind Speed Sensor 2 (m/s)
Ambient Temperature (${}^{\circ }C$)
Rotor Speed (rpm)
Generator Speed (rpm)
Blade Pitch (${}^{\circ }$)
Longitudinal Tower Acceleration Max 1P and 3P Frequency Amplitude (mg/s)
Transverse Tower Acceleration Max 1P and 3P Frequency Amplitude (mg/s)
Blade A, B, C Load (Nm)
Blade A, B, C Pitch Manifold Oil Pressure (bar)
Blade A, B, C Pitch Cylinder Pressure (bar)
Blade A, B, C Pitch Traveled Distance (mm)
Hydraulic Oil Pressure (bar)
Gear Main Tank Oil Pressure (bar)
Gear Inlet Oil Pressure (bar)
Generator Bearing Temperature (${}^{\circ }C$)
Gear Bearing Temperature (${}^{\circ }C$)
Hydraulic Oil Temperature (${}^{\circ }C$)

**Table 3:** List of the possible input variables for Test Case 2.

Wind Speed Sensor 1 (m/s)
Wind Speed Sensor 2 (m/s)
Rotor Speed (rpm)
Generator Speed (rpm)
Blade Pitch (${}^{\circ }$)
Maximum Drivetrain Oscillations (mg)
Blade Pitch Current Axis 1, 2, 3 (A)
Pressure Gearbox Inlet (bar)
Pressure Gear Pump (bar)
Blade Pitch Motor Temperature Blade 1, 2, 3 (${}^{\circ }C$)
Generator Bearing Temperature (${}^{\circ }C$)
Gear Bearing Temperature (${}^{\circ }C$)
Rotor Bearing Temperature (${}^{\circ }C$)
Oil Sump Temperature (${}^{\circ }C$)

**Table 4:** The selected GP model hyperparameters.

Hyperparameter	Test Case 1	Test Case 2
$\sigma_{f}^{2}$	10.1	3.7
$l^{2}$	1772.0	814.4
$\sigma_{n}^{2}$	44.6	21.5

**Table 5:** Selected input variables by SFS, Test Case 1.

Wind Speed Sensor 2
Generator Speed
Transverse Tower Acceleration Max 1P Frequency Amplitude
Blade A Manifold Oil Pressure
Longitudinal Tower Acceleration Max 1P Frequency Amplitude
Blade A Cylinder Pressure
Hydraulic Oil Temperature
Gear Main Tank Oil Pressure
Transverse Tower Acceleration Max 3P Frequency Amplitude
Blade Pitch Angle
Blade C Manifold Oil Pressure
Gear Bearing Temperature
Ambient Temperature
Blade B Manifold Oil Pressure
Blade B Cylinder Pressure

**Table 6:** Models’ Performance.

Metric	Multivariate	Univariate
MAE [−]	0.91%	1.75%
RMSE [−]	1.52%	2.44%
$\left|R_{95\%}\right|$ [−]	3.63%	5.25%
Metrics expressed in units of wind turbine rated power for Test Case 1.		

**Table 7:** List of Selected input variables by SFS, Test Case 2.

Wind Speed Sensor 1
Blade Pitch Current Axis 2
Rotor Speed
Maximum Drivetrain Oscillations
Oil Sump Temperature
Gear Pump Pressure
Gearbox Inlet Pressure
Blade Pitch Current Axis 3
Blade Pitch
Blade Pitch Current Axis 1
Rotor Bearing Temperature
Wind Speed Sensor 2

**Table 8:** Models’ Performance.

Metric	Multivariate	Univariate
MAE [−]	0.97%	2.45%
RMSE [−]	1.78%	2.81%
$\left|R_{95\%}\right|$ [−]	3.64%	7.53%
Metrics expressed in units of wind turbine rated power for Test Case 2.		

## Data Availability

The requested data is currently unavailable as the owner has deemed it confidential for economic reasons.
